# Review of deep learning models with Spiking Neural Networks for modeling and analysis of multimodal neuroimaging data

**DOI:** 10.3389/fnins.2025.1623497

**Published:** 2025-11-14

**Authors:** Ayesha Khan, Vickie Shim, Justin Fernandez, Nikola K. Kasabov, Alan Wang

**Affiliations:** 1 Auckland Bioengineering Institute, The University of Auckland, Auckland, New Zealand; 2 Department of Engineering Science and Biomedical Engineering, The University of Auckland, Auckland, New Zealand; 3 Knowledge Engineering and Discovery Research Institute, Auckland University of Technology, Auckland, New Zealand; 4 Medical Imaging Research Center, Faculty of Medical and Health Sciences, The University of Auckland, Auckland, New Zealand; 5 Centre for Co-Created Aging Research, The University of Auckland, Auckland, New Zealand; 6 Centre for Brain Research, The University of Auckland, Auckland, New Zealand

**Keywords:** neuroimaging, multimodalities, deep learning, machine learning, spiking neurons, Spiking Neural Networks, functional MRI, structural MRI

## Abstract

Medical imaging has become an essential tool for identifying and treating neurological conditions. Traditional deep learning (DL) models have made tremendous advances in neuroimaging analysis; however, they face difficulties when modeling complicated spatiotemporal brain data. Spiking Neural Networks (SNNs), which are inspired by real neurons, provide a promising option for efficiently processing spatiotemporal data. This review discusses current improvements in using SNNs for multimodal neuroimaging analysis. Quantitative and thematic analyses were conducted on 21 selected publications to assess trends, research topics, and geographical contributions. Results show that SNNs outperform traditional DL approaches in classification, feature extraction, and prediction tasks, especially when combining multiple modalities. Despite their potential, challenges of multimodal data fusion, computational demands, and limited large-scale datasets persist. We discussed the growth of SNNs in analysis, prediction, and diagnosis of neurological data, along with the emphasis on future direction and improvements for more efficient and clinically applicable models.

## 1 Introduction

Medical imaging is a fundamental and widely used tool for diagnosing various diseases and planning their treatments in the medical field. Different medical imaging techniques, including X-ray, ultrasound, digital mammography, computed tomography (CT), magnetic resonance imaging (MRI), positron emission tomography (PET), and digital pathology, are used to produce images (Pham et al., 2000; Tsuneki, 2022). In neuroscience, multimodal neuroimaging techniques, including functional MRI (fMRI), structural MRI (sMRI), diffusion tensor imaging (DTI), and others provides distinct yet interrelated aspects of neural anatomy and activity. Integrating these modalities enables a more comprehensive understanding of neural mechanisms and disease processes. Furthermore, the healthcare sector focuses on adopting computer-assisted tools to increase diagnostic accuracy and efficiency because of the advancements in artificial intelligence and neuroimaging techniques. These AI-based systems facilitate real-time disease prediction and thoroughly evaluate treatment options (Tsuneki, 2022; Yang and Yu, 2021).

Rapid progress in artificial intelligence (AI) and machine learning has further advanced neuroimaging, especially through deep learning (DL) techniques that automate the analysis of complex, high-dimensional brain data. Compared with conventional machine learning methods, DL can handle high-dimensional neuroimaging datasets with minimal manual preprocessing. As a result, multiple tasks like disease classification, predictive modeling, and the visualization of brain structure and function can be achieved more efficiently and with increased productivity (Yan et al., 2022). These advances have been supported by the growing availability of large-scale datasets and increased computational power (Calhoun et al., 2014).

However, most existing DL approaches rely on static or single modality data, which limits their ability to capture the complex patterns across space and time of the human brain (Plis et al., 2014). This gap highlights the requirement of the models that are not only data-driven but also biologically interpretable Spiking Neural Networks (SNNs) effectively transform neuroimaging and help diagnose neurological disorders by stimulating the brain’s natural processing. This makes SNN highly effective for spatiotemporal data analysis. SNN enables early detection of conditions like dementia and predicts epileptic seizures by identifying complex EEG patterns. Additionally, integrating SNN with multimodal neuroimaging, such as EEG and MRI, can enhance diagnostic accuracy by highlighting their transformative potential in neurological healthcare (Kasabov, 2019).

This study aims to critically evaluate the role of SNNs in multimodal MRI analysis and assess their potential to overcome the limitations of traditional deep learning models. The review will explore:

1. How have SNNs been applied to neuroimaging, particularly multimodal MRI data?
2. What are the advantages of SNNs over conventional deep learning models in capturing spatio-temporal features?
3. How can SNNs integrate multiple MRI modalities to enhance diagnostic accuracy?
4. What are the current challenges in implementing SNNs for large-scale multimodal MRI analysis?

By answering these questions, the review aims to highlight the strengths, limitations, and clinical potential of SNNs in neuroimaging and the findings will contribute to the advancement of biologically inspired deep learning models for more accurate, interpretable, and clinically relevant neuroimaging solutions.

### 1.1 Background

Over the past decade, deep learning (DL) models, including convolutional neural networks (CNNs), recurrent neural networks (RNNs), and long short-term memory (LSTM) networks, have transformed neuroimaging research. These models excel at extracting hierarchical features from brain images and temporal sequences, enabling improvements in classification and disease prediction (Yan et al., 2022; Yang and Yu, 2021). However, the dynamic nature of brain data poses challenges for traditional deep learning models, especially basic feedforward architectures. Their primary shortcoming is that they are unable to efficiently learn the temporal relationships that exist in spatiotemporal neurons due to a lack of internal state or memory. More importantly, their continuous, rate-based functioning is not well suited to mimic the sparse, event-driven communication of real neurons, where information is frequently contained in the exact timing of discrete spikes. Table 1 below represents a small conceptual comparison of other Deep Learning (DL) and Spiking Neural Networks (SNN) models for the most relevant aspects.

**TABLE 1:** Conceptual overview comparing deep learning (DL) and Spiking Neural Networks (SNN).

Aspect	Deep learning	Spiking Neural Networks
Information type	Continuous activation	Discrete spike events
Temporal modeling	Limited	Strong temporal dynamics
Biological realism	Low	High (mimics neuron firing)
Energy efficiency	High computational cost	Neuromorphic efficiency
Neuroimaging relevance	Good for static data	Effective for spatiotemporal data

Spiking Neural Networks (SNNs) process information through discrete spikes, mimicking the temporal firing patterns of biological neurons (Izhikevich, 2006). This spike-based communication allows SNNs to model time-dependent and event-driven brain dynamics more effectively. Especially for neuroimaging, this biologically plausible computation offers a promising bridge between neuroscience and AI, making the way for interpretable and energy-efficient models (Kasabov, 2019). Traditional deep learning models use continuous mathematical functions to represent neuron activations, whereas spiking neural networks transmit information through discrete spike events that occur over time. This difference gives SNNs a temporal dimension that is absent in most deep learning models. While CNNs and RNNs can provide efficiency in spatial or sequential pattern recognition, in contrast, SNNs capture both simultaneously, enabling effective modeling of dynamic brain processes (Ghosh-Dastidar and Adeli, 2009; Kasabov et al., 2016). Moreover, SNNs have the potential for low-power and neuromorphic hardware implementation, making them especially suitable for real-time neuroimaging analysis (Ghosh-Dastidar and Adeli, 2009).

## 2 Literature review methodology

Preferred Reporting Items for Systematic Reviews and Meta-Analyses (PRISMA) guidelines are used for conducting this review (Moher et al., 2009). The primary purpose of conducting this literature review is to analyze the trends and landscape of current advancements in deep learning models with SNN for multimodal neuroimaging data analysis.

Multiple databases are used to extract articles for this study, including PubMed, IEEE Xplore, ScienceDirect, Scopus, and Nature. A few of the papers were added from other internet sources. The main keywords used are “Neuroimaging,” “Spiking Neural Networks,” and “Deep Learning,” refined with Boolean operators for accuracy. Ten years of studies are used to retrieve relevant data (ranging from 2015 to 2025 for thematic study) to ensure the inclusion of comprehensive coverage of the knowledge and up-to-date information, as fewer relevant studies have been found in recent years.

Article selection followed PRISMA guidelines, with inclusion and exclusion determined through a three-phase process. In the identification section, all duplications are removed. During the screening phase, the title and abstract are used to identify articles that are irrelevant for exclusion and those that are relevant for inclusion. Afterward, the full text is used to extract the most relevant studies in this review. Full inclusion criteria and the list of keywords are provided in the Supplementary material.

## 3 Search results

**FIGURE 1:**
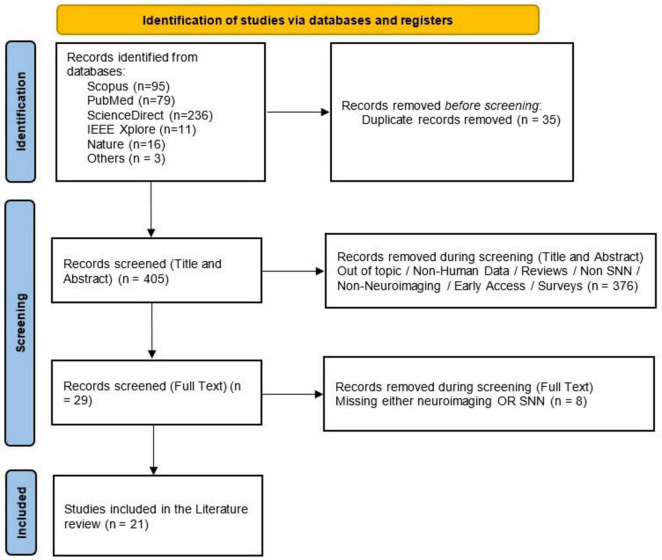
Overview of the PRISMA flowchart for this research representing the complete process followed for selecting articles for this review paper for quantitative and qualitative analysis.

Firstly, for conducting this research, the query was executed on March 10, 2025, considering the three main topics mentioned in the above section to align the study’s objectives. As a result of the query, 440 papers were retrieved from multiple databases, along with three articles from other sources. The PRISMA flowchart in Figure 1 illustrates the exclusion criteria applied to articles searched in each phase. After applying the exclusion criteria to the selected dataset in each phase, only 21 articles were chosen for this literature review.

### 3.1 Quantitative analysis

This section analyzes 21 selected articles categorized into three main perspectives quantitatively. Firstly, the study represents the annual publication trends focusing on tracking the progress of SNNs in healthcare. Secondly, research field analysis includes the articles’ distribution across various study domains. Lastly, geographical publication trends highlight contributions from different parts of the world.

#### 3.1.1 Annual publication trends: tracking progress in SNN adoption for healthcare

**FIGURE 2:**
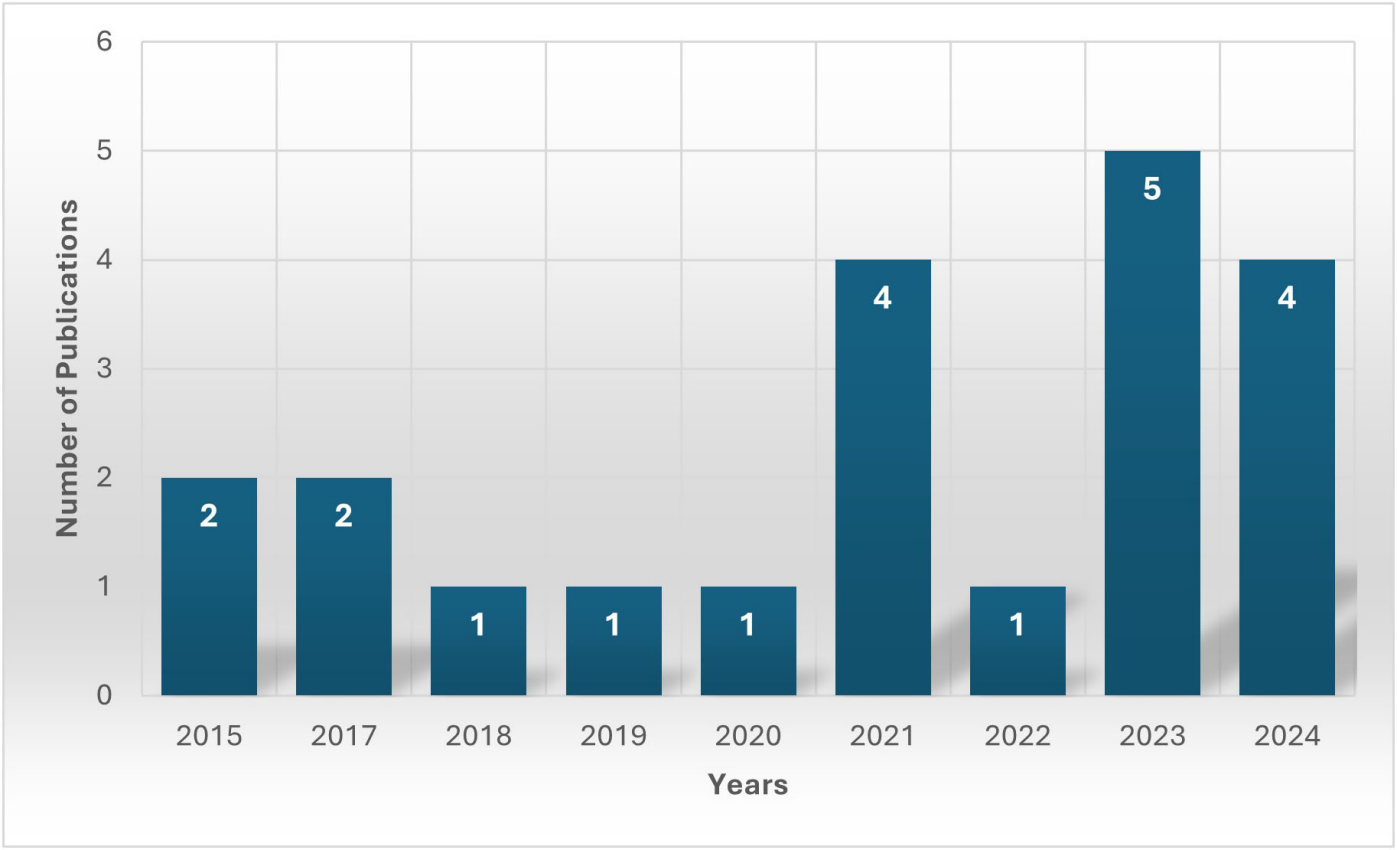
Overview of the annual publication trend, *X*-axis shows the years whereas the *Y*-axis represents the total number of publications (present on the bars) each year from the selected studies.

The analysis of 21 selected publications highlights the evolving application of Spiking Neural Networks (SNNs) in neuroimaging over the past decade, as shown in Figure 2. The citations of all 21 publications were collectively analyzed, as presented in Table 2.

**TABLE 2:** Chronological overview of citations of the selected studies.

Years	No. of publications	Total citations	Citations ≤ 20	>20 & ≤50 citations	>50 & ≤100 citations	Average citations per year
2015	2	49	1	1	0	**24.5**
2017	2	100	0	1	1	**50**
2018	1	25	0	1	0	**25**
2019	1	12	1	0	0	**12**
2020	1	0	1	0	0	**0**
2021	4	128	2	2	0	**32**
2022	1	21	0	1	0	**21**
2023	5	49	4	1	0	**8.17**
2024	4	9	1	0	0	**2.25**

The bold values represent the average annual citations of the studies.

States reveal that, from 2015 to 2017, this area of study was in the early exploration phase, as only two publications from the selected studies were available each year. Moreover, the trend shows the same results for 2018, 2019 and 2020, limiting studies to just one article per year from the chosen dataset. The reason for this decline could be methodological challenges.

However, there was a noticeable rise in research in 2021 with four publications, suggesting increasing feasibility with advancements in deep learning. After a drop in 2022 to only one study (which could be due to the selected dataset), results showed a surge in 2023 in this research area, with five studies marking a significant shift toward SNN applications. The most probable reason for this growth could be the improvements in computational resources and model efficiency.

However, the publication trend declined to four in the year 2024, still reflecting that this research area is reaching stability and reflecting the growing integration of SNNs in neuroimaging. Overall, the research has progressively grown from initial exploration to more practical implementation and refinement, which reflects a growing confidence in the field.

#### 3.1.2 Research field analysis

**FIGURE 3:**
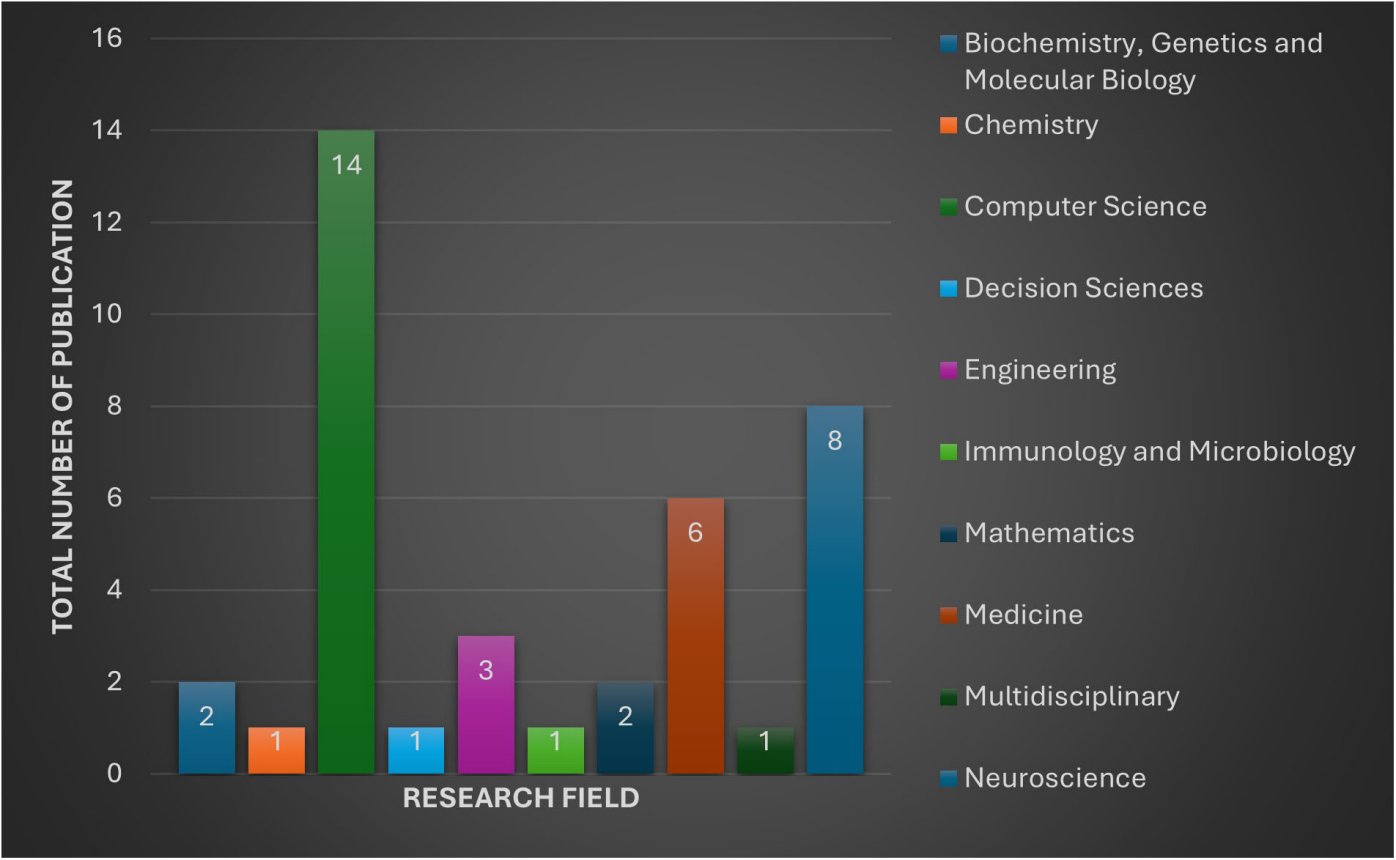
The figure provides an overview of 10 distinctive research fields and number of publications in each subject, selected for this review.

Based on classifications from the selected databases, the chosen articles are linked with 10 distinctive research fields, with some spanning multiple areas. In total, 40 connections have been established between the 21 publications and these research domains. As depicted in Figure 3, many publications fall within “Computer Science” (*n* = 14), followed by “Neuroscience” (*n* = 8) and “Medicine” (*n* = 6).

From the perspective of the biomedical research field, a collective contribution is found to make it a multidisciplinary nature of studies, such as “Medicine” (*n* = 6), “Biochemistry, Genetics, and Molecular Biology” (*n* = 2), and “Immunology and Microbiology” (*n* = 1).

From an engineering and computational standpoint, “Computer Science” (*n* = 15) and “Engineering” (*n* = 3) reflect a major division of the associations.

Additionally, research contributions span other diverse fields, including “Mathematics” (*n* = 2), “Decision Sciences” (*n* = 1), “Multidisciplinary” (*n* = 1), and “Chemistry” (*n* = 1), further highlighting the interdisciplinary scope of the selected publications.

#### 3.1.3 Geographical publication trends

In terms of geographical trend, each publication has been linked to the countries affiliated with its authors. The study of 21 selected publications reveals contributions from multiple countries. A single paper can be linked to one or multiple countries, as each publication might have multiple authors, and each author can be affiliated with more than one country. However, if a paper has multiple authors from the same country, we only consider it once to avoid duplication.

**FIGURE 4:**
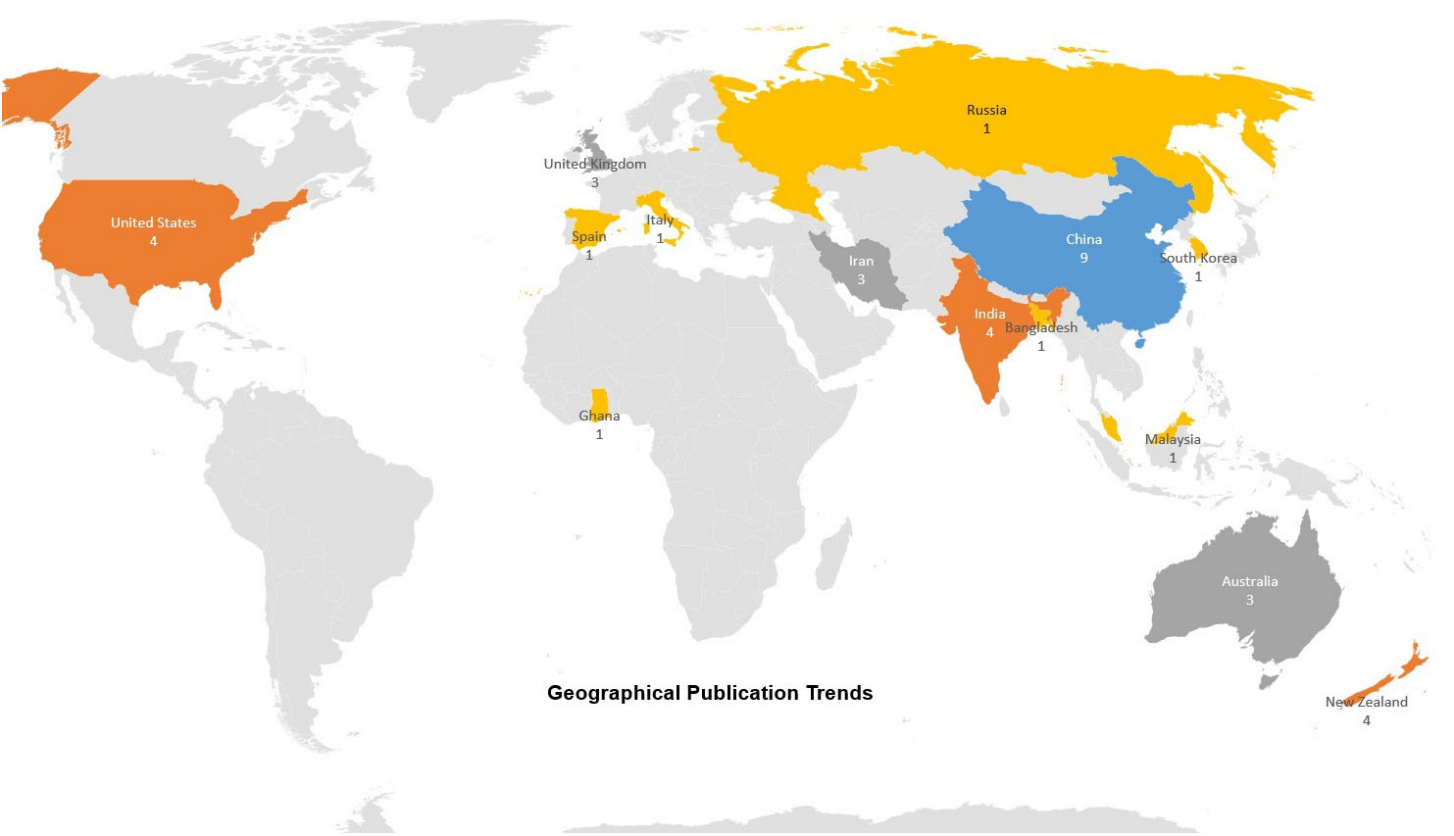
An overview of publication trends by geography highlights both the number of publications and the countries where the authors are based.

At the point of analysis, 10 countries have contributed to at least one publication. Figure 4 below categorizes the publications into single-country papers and international collaboration papers, highlighting the involvement of different countries.

China is the most frequent among the contributing countries, participating in 8 publications from the selected studies. Afterward, the highest contribution is found from the authors from New Zealand and India, each contributing to 4 publications. The United States has been involved in 3 publications. Regarding international collaboration, contributions were found in this area of research from the United Kingdom, Iran, Australia, and New Zealand. Other contributing countries include Malaysia, Russia, Ghana, Spain, Bangladesh, South Korea, and Italy, all of whom have participated in at least one publication.

Below is the comparison between a country’s contributions vs. international collaboration: -

- Results of single-country publications: 10 out of 21 papers (49%) were found to have authors solely from one country.
- Results of international collaboration publications: 11 out of 21 papers (51%) involved authors from multiple countries, demonstrating a strong trend of global cooperation in this study area.

China is the most active in single-country and collaborative research, as signified by its leadership in this field from the selected studies. New Zealand and the United Kingdom are heavily engaged in international collaborations, reflecting their strong global research attitude and commitment to cross-border partnerships. India and China are leading in solely conducted research, showcasing their independent research contributions and advancements in the field. Notably, the United States appears only in collaborative research in our selected dataset. It suggests that the US primarily participates in multinational research projects rather than independent studies.

### 3.2 Thematic analysis of selected studies

This section provides a detailed insight into selected studies for the literature review. Neuroimaging, multimodal data analysis, utilization of spiking neural networks, and clinical applications are the focus of this review section.

#### 3.2.1 Neuroimaging and multimodal data analysis

Healthcare professionals have used multiple types of medical data, including biomedical data such as electroencephalography (EEG), electromyographic signal (EMG), electrocardiogram (ECG), ultrasound, X-rays, computed tomography and magnetic resonance imaging (MRI), for a long time to judge patients’ diseases, diagnoses, and health conditions. Neuroimaging techniques are one of the various types for dealing with a wide range of conditions in the brain. MRI has recently been the most used technique, and it is said to be a vital technique for diagnosing brain tumors (Ahmadi et al., 2021; Sam et al., 2023) because it can generate images without damaging brain tissues (Dong et al., 2024). Furthermore, Kamal et al. (2023) used MRI to identify microbleeds in the brain, including the gene expression data, making it a multimodal analysis for determining the severity of Alzheimer’s disease.

In addition to traditional imaging techniques, other non-invasive methods such as functional magnetic resonance imaging (fMRI), diffusion tensor imaging (DTI), and electroencephalography (EEG) have been used for brain data collection in the recent past. These techniques played a dynamic role in helping us understand the functional and structural characteristics of the human brain in a better way (Sengupta et al., 2018). Capecci et al. (2015b) presented that EEG spatiotemporal data were used to study brain pathology and degeneration to analyze the functional changes in the brain activities of the controlled and Alzheimer’s disease (AD) groups. Furthermore, Kasabov N. K. et al. (2017) used fMRI in their research to develop a methodology using the NeuCube architecture of spiking neural network (SNN) for visualization, classification, and dynamic learning for spatiotemporal brain data. Guo et al. (2023) also applied fMRI like (Kasabov N. K. et al., 2017) for their study based on a fMRI based SNN for verifying anti-damage capabilities under random attacks.

In addition to the above-mentioned diseases, Sam et al. (2023) presented the use of EEG signal data for the quantitative assessment of depression levels by examining and categorizing EEG signals, and Francis and Al-Hababi (2023) utilized structural magnetic resonance images (sMRI) for early detection of suicidal ideation in youngsters and used depression as a biomarker in sMRI, respectively.

Most studies used a single or a few modalities to analyze different areas. However, combining predictive modeling with multimodal brain data has great potential; research in this area is still in its early stages due to a lack of advanced methods. The major challenge in combining brain data into a single model captured from various modalities is that each modality uses different temporal and spatial characteristics. To address this, the recent advancement in Spiking neural network systems (SSNs) makes it possible to incorporate multidimensional data within a single model (Sengupta et al., 2018).

#### 3.2.2 Integration of deep learning models with SNNs for neuroimaging

Spiking neural networks are one of the most reliable techniques that computer simulations and computational models use to study the brain, e.g., brain-inspired machine learning. These techniques can reveal and learn frequency, time, and space information usually hidden in spatiotemporal brain data (STBD) Capecci et al. (2015a), Sengupta et al. (2018) presented an approach integrating multimodal information with a spiking neural network framework, creating a personalized SNNc-based architecture using the NeuCube. This experiment was run to represent the algorithm’s capabilities (oiSTDP) to capture discriminative join information from the data through its connection strengths. Utilizing this framework, the study showcases the integration of DTI and fMRI data of individuals who are beginning antipsychotic treatment to develop a personalized classifier for the prediction of treatment response in schizophrenia. Analysis with the SNNc network uncovered improved connectivity in the cerebellar region, which suggests that the captured activity of this area can be an aid as a potential biomarker that would help in treatment response in individuals with schizophrenia.

**FIGURE 5:**
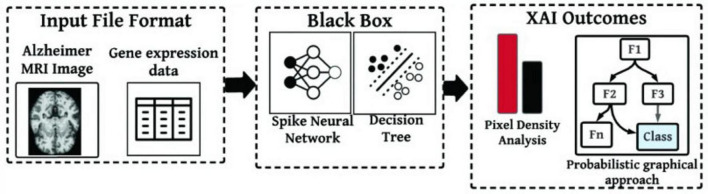
A framework to identify cerebral microbleeds and Alzheimer from multimodal data (Kamal et al., 2023).

Kamal et al. (2023) stated that machine learning techniques are practical for analyzing Alzheimer’s disease datasets. Machine learning techniques can predict the disease by detecting microbleeds in the brain. The study used MRI images and gene expression data, making it multimodal for identifying microbleeds using SNN and decision trees. Afterward, pixel density analysis (PDA) was used to locate microbleed areas in MRI images. PDA and probabilistic graphical model (PGM) were also used to explain and decide on the diagnosis of microbleeds, and the severity of AD. Figure 5 represents the framework used in this research for identifying cerebral microbleeds and Alzheimer’s using multimodal data.

Furthermore, the study compared SNN with other state-of-the-art methods for performance evaluation using recall, accuracy, precision, and F-score. Table 3 below shows that SNN yielded the highest performance with 96.13%, 97.02%, 97.13%, and 96.84% for recall, accuracy, precision, and F-score, respectively.

**TABLE 3:** Performance evaluation for SNN and CNN (Kamal et al., 2023).

Method	Training	Test	PREC	REC	ACC	F-score
SNN	552	98	97.13	96.13	97.02	96.84
	520	1300	96.23	94.33	97.12	95.14
	487	163	95.19	96.23	96.82	96.04
	455	195	94.03	95.03	95.62	95.14
CNN	552	98	91.53	93.43	94.61	94.05
	520	130	91.47	92.45	92.24	92.46
	487	163	91.19	94.39	92.53	93.24
	455	195	92.73	91.03	91.92	91.47

**FIGURE 6:**
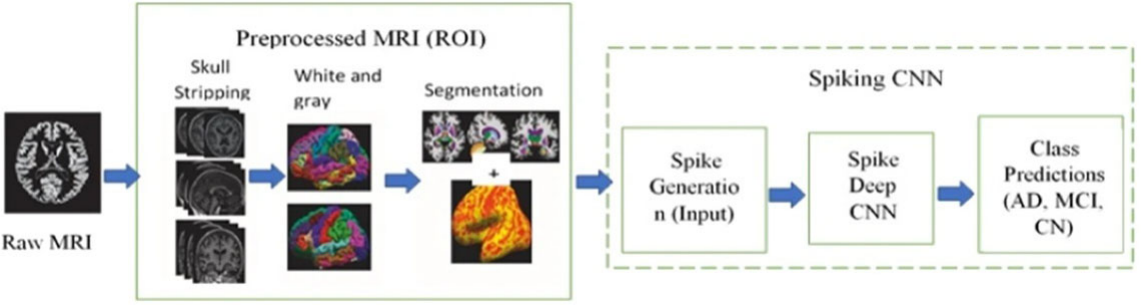
An overview of the proposed hybrid model, called the spiking deep convolutional neural network, is shown in the figure. It highlights three main modules: raw data input, preprocessing, and the spiking model (Turkson et al., 2021).

Whereas Turkson et al. (2021) proposed a hybrid model, a spiking deep convolutional neural network architecture to classify MRI images to detect Alzheimer’s disease. Figure 6 shows the general architecture proposed in this study. The Alzheimer’s disease neuroimaging initiative (ADNI) dataset (450 scans) was used to perform three binary classification tasks (AD vs. NC, AD vs. MCI, and NC vs. MCI). The pre-processed images were used to extract AD key features using an unsupervised spiking neural network. The pre-trained spikes were then classified using a supervised deep convolutional neural network (CNN). The SNN was tested with two models, firstly with a model that was pre-trained with a spike, and secondly with a model without a pre-trained spike, and the results were more accurate with a pre-trained model for three binary classification tasks. The study stated that SNN improved performance, demonstrating the potential of spiking networks for reliable neuroimaging analysis.

Moreover, Doborjeh et al. (2021) used longitudinal neuroimaging data to utilize the advancements in deep learning models through brain-inspired spiking neural networks (SNNs), which can model structural brain data across time and space. The study introduced a methodology and an SNN-based computational framework for developing personalized, predictive models from longitudinal brain data to detect, interpret, and predict changes in an individual’s functional brain state. The approach consists of several steps, such as clustering similar data, incorporating missing values, and training a 3D brain-template SNN for classifying and predicting outcomes and visualizing structural brain changes. All this information is then used to interpret results and identify predictive markers at both individual and group levels. As a result, the model successfully classified and predicted cognitive decline, for example, dementia and mild cognitive impairment (MCI), 2 years ahead with 91% and 95% accuracy.

#### 3.2.3 Clinical applications

Previous sections primarily focused on the types of modalities used by several researchers, along with the integration of machine learning models using SNN to analyze neuroimaging data (Doborjeh et al., 2019; Saeedinia et al., 2021; Ghazali et al., 2020). Moreover, multiple studies highlight practical applications using neuroimaging and SNNs for several diseases, such as the detection of AD and the prediction of schizophrenia. However, neuroimaging, multimodal data analysis, and integration of deep learning with spiking neural networks are not only vital for AD but also for many other clinical areas, such as brain tumor classification (Kalpana et al., 2024) or segmentation (Ahmadi et al., 2021), suicide ideation assessment (Francis and Al-Hababi, 2023), Depression identification (Sam et al., 2023), Parkinson’s disease detection (Das et al., 2024), and so on.

Kalpana et al. (2024) proposed a hybrid model integrating SNNs with convolutional neural networks (CNNs) for brain tumor classification. As per the results, the model achieved a testing accuracy of 97.50 % and a training accuracy of 97.79% with a slight loss value of 0.38%. Also stated, these results present the model’s strength in capturing complex data in medical imaging, which is promising for clinical applications within the biomedical field. On the other hand, Ahmadi et al. (2021) utilized MRI for tumor area segmentation using a deep spiking neural network. The process for achieving the results consists of preprocessing and segmentation using DSNN. Table 4 shows the accuracy percentages compared in this study for the proposed method and previous approaches.

**TABLE 4:** Performance evaluation for QAIS-DSNN and other methods (Ahmadi et al., 2021).

Accuracy (%)	Method	Reference
98.20%	GCNN	Mittal et al., 2019
95%	3D cascaded CNN-TTA	Wang et al., 2019
88.50%	MCCNN	Hu et al., 2019
96.12%	BAT-IT2FCM	Alagarsamy et al., 2019
92%	ADNN-PSO	Sharif M. I. et al., 2020
98%	PSO-LDA-GA-ANN	Sharif M. et al., 2020
98.21%	QAIS-DSNN	Proposed approach (Ahmadi et al., 2021)

Furthermore, Francis and Al-Hababi (2023) also incorporated a hybrid version like Kalpana et al. (2024). However, they combined an attention mechanism (AM) with SNN for suicide ideation assessment using sMRI of healthy controls and depressive individuals who did not show any suicide ideation (SI). The hybrid model completed the classification tasks using stratified 5-fold cross-validation and achieved a test accuracy of 94%, sensitivity of 100%, specificity of 92%, and an area under the curve (AUC) of 0.96. The proposed algorithm provides an objective tool that can support clinical assessments in identifying early signs of SI risk among depressed patients who are currently not showing any suicidal thoughts.

Sam et al. (2023) performed a similar study to Francis and Al-Hababi (2023) by combining long short-term memory (LSTM) and SNN using EGG signals for depression identification. These models were used for the first time to analyze and categorize EEG signals according to the level of depression (minimum, mild, moderate, and severe). The model utilized spatial data mapping using SNN, leading to unsupervised learning and visualization of spiking patterns and unique perceptions about brain mechanisms. Comparative analysis of time-space brain data showed that SNN is more advantageous than other deep learning models. The study achieved higher accuracy in classifying samples from various groups and disclosed different patterns of brain activity, helping to understand the severity of depression. The findings in the study have the potential for early prediction and, thus, preventing depression by using the brain data acquired from different depression levels. The methodology used in this study showcases excellent potential for application in neuroimaging and clinical longitudinal data.

Apart from the above-mentioned clinical applications, SNN and neuroimaging are also helpful for Parkinson’s disease (PD) detection, as presented by Das et al. (2024). This study investigated the SEFRON (time-varying synaptic efficacy function based leaky integrate and fire neuron model) model and compared it with other previously used neural network models such as RBF, RNN, LSTM, and MLP. Results showed a higher accuracy for this model over others, making it reliable for clinical trials. Moreover, because of its performance, this model can help develop an automated PD detection device that physicians can utilize to diagnose PD in its early stages. However, this study has a limitation in that the sample size of the dataset used is small.

Another study proposed by Gowsikraja et al. (2025) uses MRI for AD classification using a model named SBERO_Deep SNN (skill Al-Biruni Earth Radius Optimization-enabled Deep Spiking Neural Network), encompassing the benefits of SNN. Segmentation was performed using a hybrid algorithm using UNeXt, combining the Skill Optimization Algorithm (SOA) and the Al-Biruni Earth Radius (BER). In the next step, statistical features were extracted and classified using a Deep SNN trained with SBERO. The study achieved the highest accuracy rate compared to other AD classification techniques. It also states that the proposed methodology is reliable for AD identification, allowing timely intervention to slow the disease progression and improve patient quality of life. Table 5 represents the overview of selected studies below.

**TABLE 5:** Overview of selected literature.

References	Models used	Modalities	Compared with	Accuracy %/ performance
Ahmadi et al., 2021	QAIS-DSNN	MRI	KNN, Genetic algorithm, SVM, SOM, CNN, GCNN, BAT-IT2FCM	DSNN with 98.21 %
Dong et al., 2024	ONSNPSamos	MRI	ABC, CMA-ES, SHADE, CSO, RMSProp, FROFI, DAOSNPS, ONSNPS, FCN8s, FCNs16, FCNs32, Unet, HybridUnet, NestedUnet	Effective for a Single-area brain tumor
Kamal et al., 2023	SNN	MRI & Gene expression	CNN	97.02%
Sengupta et al., 2018	SNN	DTI & fMRI	Not applicable	Effective
Capecci et al., 2015b	SNN	EEG	MLP, SVM, IECF, ECMC	100%
Capecci et al., 2015a	SNN (NeuCube)	EEG	MLR, SVM, MLP, ECM	86%, 79%
Kasabov N. et al., 2017	SNN (NeuCube)	fMRI	SVM, MLP, ECF, ECMC, MLR	90%, 85%, 85%
Guo et al., 2023	SNN	fMRI	SFSNN, SWSNN	Outperformed
Sam et al., 2023	SNN	EEG	CNN-TCN, CNN-LSTM, Deep CNN	98%: 96%, Eyes-Closed: Eyes-Open
Francis and Al-Hababi, 2023	SNN	sMRI	CNN, SVM, FCNN	94%
Turkson et al., 2021	Spiking deep convolutional neural network	MRI	SVM, CNN, random forest, KNN, NB	90.15%
Doborjeh et al., 2021	SNN	Longitudinal MRI	Not applicable	95% & 91%
Doborjeh et al., 2019	Personalized SNN - d2WKNN	EEG	WWKNN, WKNN, KNN	80% to 93%
Saeedinia et al., 2021	MRI-SNNr	MRI, EEG	Random walk, NeuCube	Better prediction
				accuracy
Murli et al., 2020	eSNN (NeuCube)	fMRI	SVM, MLP	85%
Kalpana et al., 2024	Hybrid SCNN	MRI	Not applicable	97.50%
Das et al., 2024	SEFRON (SNN-based)	speech measurements	RBF-NN, MLP-NN, RNN, LSTM	94% to 91.94%
		collected/acoustic features		
Kasabov N. et al., 2017	SNN (NeuCube)	fMRI	Not applicable	Compatible
Gowsikraja et al., 2025	Hybrid SBERO_Deep SNN	MRI	CNN+LSTM, Deep ensemble model, Hybrid DNN, TL, Deep SNN, SOA_Deep SNN, BER_Deep SNN	90.49%
Zhu et al., 2022	DenseNet-based SNN	MRI	Restricted DenseNet, SNN,	98.46% ± 2.05%
Dong et al., 2023	STPD-based SNN	CIFAR10 random images	MNIST, FashionMNIST	81.45%

## 4 Discussion

This section provides a detailed discussion of principal findings, including neuroimaging and analysis of clinical features and use of SNN. The section also discusses this review’s challenges, gaps, future direction, and limitations.

### 4.1 Principal findings

The primary findings of the literature review of the selected 21 studies expose a massive gap in defusing multiple modalities for developing a deep learning model using SNN for real-time clinical applications. Out of 21, only a few studies proposed the utilization of two or more modalities together for analysis. The rest of the studies focused on a single modality in their research.

The literature review reveals a significant trend toward utilizing the benefits of neuroimaging data combined with deep learning models, specifically SNN, for understanding and diagnosing neurological disorders. Researchers are applying spiking neural networks with MRI, EEG, fMRI, and DTI for personalized and dynamic modeling of brain functionalities. A comparative analysis of the selected studies on SNNs shows that SNNs provide more accuracy than other previously used methods, such as traditional CNNs, in terms of precision, recall, and accuracy. Furthermore, conventional neural networks have a significant disadvantage of slower computational speed and higher energy consumption (Xiaoxue et al., 2023).

### 4.2 Neuroimaging and clinical features

Neuroimaging, including structural MRI (sMRI), fMRI, DTI, and EEG are important in understanding the clinical features of psychiatric and neurological disorders. All these modalities provide a detailed map of brain structure, activity, and function, which is helpful for clinicians and researchers in making more accurate diagnoses and guiding the right treatments.

**FIGURE 7:**
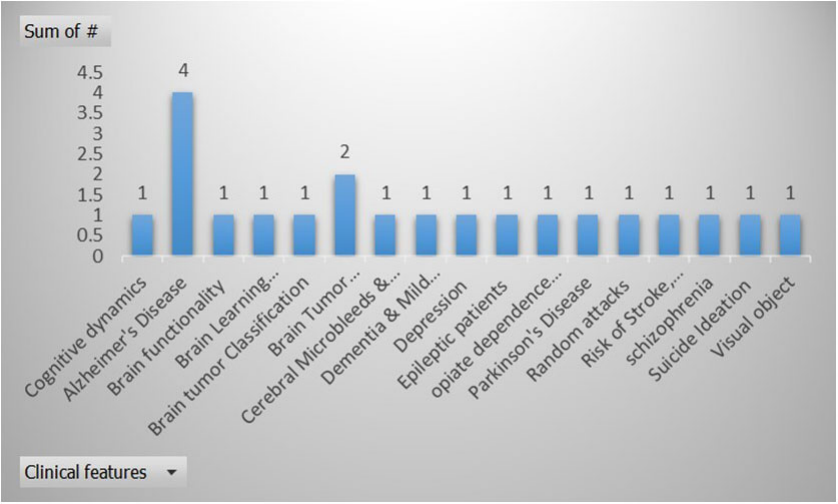
Overview of the clinical features from the selected studies, *X*-axis represents the clinical features, and *Y*-axis is used to display the number of times a clinical feature used in selected research articles.

As per the selected studies, researchers emphasize the importance of neuroimaging in identifying biomarkers such as microbleeds, structural abnormalities, and functional connectivity changes. However, using multiple modalities can enhance the depth of clinical insights. Figure 7 represents the overview of clinical features from the selected studies.

### 4.3 Use of deep learning with SNN and neuroimaging

Spiking Neural Networks (SNNs) are computational models consisting of spiking neurons, interconnections, and learning algorithms designed for processing data (Izhikevich, 2006; Kasabov et al., 2016). Unlike traditional neural networks, SNNs capture spatial and temporal data as inputs. SNN incorporates not only neural synaptic states but also the concept of time within its computational framework. This computational framework makes SNNs a more biologically realistic approach for modeling spatiotemporal brain dynamics (STBD) (Kasabov N. et al., 2017). Other than that, SNN techniques offer many benefits, including fast information processing and memory-based processing. It also supports frequency- and time-based data, allowing for post-training analysis and data interpretation (Capecci et al., 2015b).

**FIGURE 8:**
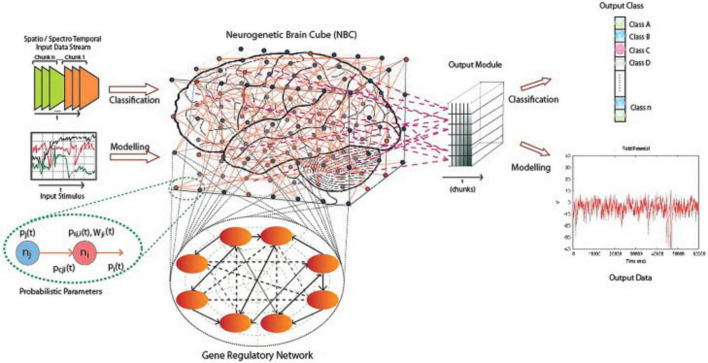
A schematic diagram of a general NeuCube architecture, consisting of, input encoding module, SNN Cube, Output module, and gene regulatory module (Kasabov, 2014).

Spiking Neural Networks (SNNs), especially implemented as NeuCube, are gaining importance due to their ability to model spatiotemporal data more efficiently and naturally (Kasabov, 2014). As aforementioned, SNNs process data in both space and time using discrete spike trains, offering an alternative that closely resembles neuronal communication in the brain, contrasting traditional artificial neural networks (ANNs) like MLPs or Transformers (Ghosh-Dastidar and Adeli, 2009; Kasabov, 2014). NeuCube provides a structured framework for developing models that process spatiotemporal brain data (STBD). It mirrors how the brain encodes and learns information through spikes, using spatial mapping and brain-inspired learning rules. The model evolves over time, continuously learning and adapting to new patterns while maintaining a spatiotemporal memory that supports the analysis of cognitive functions. The overall architecture of NeuCube is illustrated in Figure 8.

Furthermore, Kasabov et al. (2016) mentioned that the NeuCube can be used for the visualization of input features interactions at the group level as well as the individual level. It provides a more profound understanding of underlying data relationships and their impact on the individual risk of stroke.

However, this review demonstrated that SNN is also being integrated with other models, such as CNNs and LSTMs, making it a hybrid model for more efficient spatiotemporal data modeling using neuroimaging. Turkson et al. (2021), Kalpana et al. (2024) and Francis and Al-Hababi (2023) proposed a hybrid model integrating other models with SNNs using neuroimaging for the assessment of suicidal ideations, brain tumor classification, and detection of Alzheimer’s diseases, showcasing better results in terms of accuracy than other previously used deep learning models.

Likewise, SNN-based frameworks such as NeuCube (Kasabov, 2014) and other brain-inspired models make it easy to learn and utilize multiple modalities, improving accuracy even at a higher level. Capecci et al. (2015b) stated that the overall performance of NeuCube was considerably better in terms of highest precision and sensitivity than other compared classification methods, including multilayer perceptron (MLP), inductive evolving classification function (IECF) (Kasabov, 2007), support vector machine (SVM), and evolving clustering method for classification (ECMC) (Song and Kasabov, 2002). The literature review also uncovers the performance of SNNs in longitudinal studies and real-time prediction tasks, confirming their potential for future clinical applications.

Furthermore, Kundu et al. (2024) presented the recent progress in SNN algorithms and their integration with sensor and memory technologies. It underscores how SNNs enable deployable AI systems that are energy-efficient, reliable, and well-suited for real-world tasks.

## 5 Conclusion, and future research directions

As per the scope of this literature review, the aim was to incorporate neuroimaging data and SNN-based models. According to the available evidence suggests that SNN-based models may offer advantages over traditional neural networks when applied to neuroimaging data. Furthermore, these studies represent the integration of neuroimaging and deep learning models using SNN for analysis, classification, feature extraction, and diagnosis of diseases. Different types of modalities were utilized in these studies, including MRI, fMRI, DTI, and EEG. However, MRI-based modalities dominated either as a single modality or by defusing a few others. It is observed that there are only a few studies that use multimodality, either combining MRI with DTI or EEG, or with another type of data, such as gene expression. Multiple studies on Alzheimer’s disease use only a single modality. Furthermore, many studies use small or specific datasets, maybe due to the high computational power SNNs require for training. Furthermore, it is stated that the diffusion of modalities with different spatial and temporal resolutions remains a key barrier Sengupta et al. (2018). While current SNN studies have mainly focused on sMRI, fMRI, or EEG, primarily using single-modality data for Alzheimer’s disease, the study by Li and Yap (2022), which employs generative modeling, particularly for fMRI and DTI, offers a path toward mechanistic connectomes that extends beyond descriptive connectivity and explains how it captures the brain connectivity changes in response to tasks, stimuli, or internal states. The presented approach could have a significant impact on future SNN frameworks by providing better integration of multimodal data, thereby supporting improved prediction and classification accuracy.

Hence, the review suggests that exploring multiple modalities like MRI, fMRI, DTI, and perfusing MRI defusing together to develop a deep learning model using SNN for better analyses and use of clinical features for disease diagnosis, prevention, and to provide treatment plans for slower progression. Improvement in methods for multimodal data fusion is required. Moreover, large and more diverse datasets should be utilized to increase the accuracy of the models. Additionally, integrating the above-mentioned modalities with PET images can provide a critical analysis of specific disease detection, such as Alzheimer’s disease, as PET imaging techniques are a popular and non-invasive technique used to capture brain tissue characteristics, as explained by Song et al. (2021) in their study, along with the ability to directly visualize AD-specific biomarkers.

Furthermore, future studies can aim to develop a sophisticated AI partner for Alzheimer’s patients and their families or friends to help them with their diagnosis, treatment plans, reports, and daily queries. It will be helpful support for healthcare professionals in dealing with their patients. The AI model and agent should integrate all international guidelines relating to Alzheimer’s disease treatment plans and patient care.

### 5.1 Limitations

Firstly, the literature review is conducted with a few limitations, including a narrow picture of current trends in this area, as it only considers papers in English that are published in conferences or journals. This review may not cover all relevant work due to inclusion/exclusion criteria. Secondly, the review was conducted using specific keywords and databases, because of which many relevant studies might be overlooked, as limited datasets can reduce external validations.

## References

[B1] Ahmadi M. Sharifi A. Hassantabar S. Enayati S. (2021). QAIS-DSNN: Tumor area segmentation of MRI image with optimized quantum matched-filter technique and deep spiking neural network. *BioMed Res. Int.* 2021:6653879. 10.1155/2021/6653879 33542920 PMC7843186

[B2] Alagarsamy S. Kamatchi K. Govindaraj V. Zhang Y. D. Thiyagarajan A. (2019). Multi-channeled MR brain image segmentation: A new automated approach combining BAT and clustering technique for better identification of heterogeneous tumors. *Biocybernet. Biomed. Eng.* 39 1005–1035. 10.1016/j.bbe.2019.05.007

[B3] Calhoun V. D. Miller R. Pearlson G. Adalı T. (2014). The chronnectome: Time-varying connectivity networks as the next frontier in fMRI data discovery. *Neuron* 84 262–274. 10.1016/j.neuron.2014.10.015 25374354 PMC4372723

[B4] Capecci E. Kasabov N. Wang G. Y. (2015b). Analysis of connectivity in NeuCube spiking neural network models trained on EEG data for the understanding of functional changes in the brain: A case study on opiate dependence treatment. *Neural Netw.* 68 62–77. 10.1016/j.neunet.2015.03.009 26000776

[B5] Capecci E. Morabito F. C. Campolo M. Mammone N. Labate D. Kasabov N. (2015a). “A feasibility study of using the neucube spiking neural network architecture for modelling alzheimer’s disease eeg data,” in *Advances in neural networks: Computational and theoretical issues*, eds Bassis S. Esposito A. Morabito F. C. (Cham: Springer International Publishing), 159–172. 10.1007/978-3-319-18164-6_16

[B6] Das P. Nanda S. Panda G. Dash S. Ksibi A. Alsenan S. (2024). A robust Parkinson’s disease detection model based on time-varying synaptic efficacy function in spiking neural network. *BMC Neurol.* 24:492. 10.1186/s12883-024-04001-7 39734199 PMC11684134

[B7] Doborjeh M. Doborjeh Z. Merkin A. Bahrami H. Sumich A. Krishnamurthi R. (2021). Personalised predictive modelling with brain-inspired spiking neural networks of longitudinal MRI neuroimaging data and the case study of dementia. *Neural Netw.* 144 522–539. 10.1016/j.neunet.2021.09.013 34619582

[B8] Doborjeh M. Kasabov N. Doborjeh Z. Enayatollahi R. Tu E. Gandomi A. H. (2019). Personalised modelling with spiking neural networks integrating temporal and static information. *Neural Netw.* 119 162–177. 10.1016/j.neunet.2019.07.021 31446235

[B9] Dong J. Zhang G. Hu Y. Wu Y. Rong H. (2024). An optimization numerical spiking neural membrane system with adaptive multi-mutation operators for brain tumor segmentation. *Int. J. Neural Syst.* 34:2450036. 10.1142/S0129065724500369 38686911

[B10] Dong Y. Zhao D. Li Y. Zeng Y. (2023). An unsupervised STDP-based spiking neural network inspired by biologically plausible learning rules and connections. *Neural Netw.* 165, 799–808. 10.1016/j.neunet.2023.06.019 37418862

[B11] Francis C. Al-Hababi A. Y. S. (2023). “Performance evaluation of attention mechanism and spiking neural networks on smri data for suicide ideation assessment,” in *Proceedings of the 2023 IEEE International Conference on Computing*, (Langkawi: IEEE), 408–413. 10.1109/ICOCO59262.2023.10397625

[B12] Ghazali R. Nawi N. M. Deris M. M. Abawajy J. H. (2020). “Recent advances on soft computing and data mining,” in *Proceedings of the 4th International Conference on Soft Computing and Data Mining (SCDM 2020), Melaka, Malaysia, January 22–23, 2020*, (Cham: Springer International Publishing), 10.1007/978-3-030-36056-6

[B13] Ghosh-Dastidar S. Adeli H. (2009). Spiking neural networks. *Int. J. Neural Syst.* 19 295–308. 10.1142/S0129065709002002 19731402

[B14] Gowsikraja P. Geetha K. Rajan C. (2025). SBERO skill al-biruni earth radius optimization for Alzheimer’s disease classification using magnetic resonance image. *NMR Biomed.* 38:e5323. 10.1002/nbm.5323 39887547

[B15] Guo L. Liu C. Wu Y. Xu G. (2023). fMRI-based spiking neural network verified by anti-damage capabilities under random attacks. *Chaos Solitons Fractals* 176:114083. 10.1016/j.chaos.2023.114083

[B16] Hu K. Gan Q. Zhang Y. Deng S. Xiao F. Huang W. (2019). Brain tumor segmentation using multi-cascaded convolutional neural networks and conditional random field. *IEEE Access* 7 92615–92629. 10.1109/ACCESS.2019.2927433

[B17] Izhikevich E. M. (2006). Polychronization: Computation with spikes. *Neural Comput.* 18 245–282. 10.1162/089976606775093882 16378515

[B18] Kalpana T. Meghana P. Spandana M. Kumar T. S. (2024). “Biologically inspired spiking CNN for brain tumor classification,” in *Proceedings of the 2024 5th International Conference on Image Processing and Capsule Networks (ICIPCN)*, (Nepal: IEEE), 10.1109/ICIPCN63822.2024.00035

[B19] Kamal M. S. Chowdhury L. Nimmy S. F. Hasan Rafi T. H. Chae D. K. (2023). An interpretable framework for identifying cerebral microbleeds and Alzheimer’s disease severity using multimodal data. *Annu. Int. Conf. IEEE Eng. Med. Biol. Soc.* 2023 1–4. 10.1109/EMBC40787.2023.10340088 38082672

[B20] Kasabov N. Scott N. M. Tu E. Marks S. Sengupta N. Capecci E. (2016). Evolving spatio-temporal data machines based on the NeuCube neuromorphic framework: Design methodology and selected applications. *Neural Netw.* 78 1–14. 10.1016/j.neunet.2015.09.011 26576468

[B21] Kasabov N. Zhou L. Doborjeh M. G. Doborjeh Z. G. Yang J. (2017). New algorithms for encoding, learning and classification of fMRI data in a spiking neural network architecture: A case on modeling and understanding of dynamic cognitive processes. *IEEE Trans. Cogn. Dev. Syst.* 9 293–303. 10.1109/TCDS.2016.2636291

[B22] Kasabov N. K. (2007). *Evolving connectionist systems: The knowledge engineering approach*, 2nd Edn. London: Springer.

[B23] Kasabov N. K. (2014). NeuCube: A spiking neural network architecture for mapping, learning and understanding of spatio-temporal brain data. *Neural Netw.* 52 62–76. 10.1016/j.neunet.2014.01.006 24508754

[B24] Kasabov N. K. (2019). *Time-Space, spiking neural networks and brain-inspired artificial intelligence.* Berlin: Springer.

[B25] Kasabov N. K. Doborjeh M. G. Doborjeh Z. G. (2017). Mapping, learning, visualization, classification, and understanding of fMRI data in the neucube evolving spatiotemporal data machine of spiking neural networks. *IEEE Trans. Neural Netw. Learn. Syst.* 28 887–899. 10.1109/TNNLS.2016.2612890 27723607

[B26] Kundu S. Zhu R. J. Jaiswal A. Beerel P. A. (2024). “Recent advances in scalable energy-efficient and trustworthy spiking neural networks: From algorithms to technology,” in *Proceedings of the ICASSP 2024-2024 IEEE International Conference on Acoustics, Speech and Signal Processing (ICASSP)*, (Korea: IEEE), 10.1109/ICASSP48485.2024.10445826

[B27] Li G. Yap P. T. (2022). From descriptive connectome to mechanistic connectome: Generative modeling in functional magnetic resonance imaging analysis. *Front. Hum. Neurosci.* 16:940842. 10.3389/fnhum.2022.940842 36061504 PMC9428697

[B28] Mittal M. Goyal L. M. Kaur S. Kaur I. Verma A. Hemanth D. J. (2019). Deep learning based enhanced tumor segmentation approach for MR brain images. *Appl. Soft Comp.* 78 346–354. 10.1016/j.asoc.2019.02.036

[B29] Moher D. Liberati A. Tetzlaff J. Altman D. G. (2009). Preferred reporting items for systematic reviews and meta-analyses: The PRISMA statement. *PLoS Med.* 6:e1000097. 10.1371/journal.pmed.1000097 19621072 PMC2707599

[B30] Murli N. Kasabov N. Paham N. A. (2020). “eSNN for Spatio-temporal fMRI brain pattern recognition with a graphical object recognition case study,” in *Recent advances on soft computing and data mining. SCDM 2020. Advances in intelligent systems and computing*, vol 978, eds. Ghazali R. Nawi N. Deris M. Abawajy J. (Cham: Springer). 10.1007/978-3-030-36056-6_44

[B31] Pham D. L. Xu C. Prince J. L. (2000). Current methods in medical image segmentation. *Annu. Rev. Biomed. Eng.* 2 315–337. 10.1146/annurev.bioeng.2.1.315 11701515

[B32] Plis S. M. Hjelm D. R. Salakhutdinov R. Allen E. A. Bockholt H. J. Long J. D. (2014). Deep learning for neuroimaging: A validation study. *Front. Neurosci.* 8:229. 10.3389/fnins.2014.00229 25191215 PMC4138493

[B33] Saeedinia S. A. Jahed-Motlagh M. R. Tafakhori A. Kasabov N. (2021). Design of MRI structured spiking neural networks and learning algorithms for personalized modelling, analysis, and prediction of EEG signals. *Sci. Rep.* 11:12064. 10.1038/s41598-021-90029-5 34103545 PMC8187669

[B34] Sam A. Boostani R. Hashempour S. Taghavi M. Sanei S. (2023). Depression identification using EEG signals via a hybrid of LSTM and spiking neural networks. *IEEE Trans. Neural Syst. Rehabil. Eng.* 31 4725–4737. 10.1109/TNSRE.2023.3336467 37995160

[B35] Sengupta N. McNabb C. B. Kasabov N. Russell B. R. (2018). Integrating space, time, and orientation in spiking neural networks: A case study on multimodal brain data modeling. *IEEE Trans. Neural Netw. Learn. Syst.* 29 5249–5263. 10.1109/TNNLS.2018.2796023 29994642

[B36] Sharif M. Amin J. Raza M. Yasmin M. Satapathy S. C. (2020). An integrated design of particle swarm optimization (PSO) with fusion of features for detection of brain tumor. *Pattern Recogn. Lett.* 129 150–157. 10.1016/j.patrec.2019.11.017

[B37] Sharif M. I. Li J. P. Khan M. A. Saleem M. A. (2020). Active deep neural network features selection for segmentation and recognition of brain tumors using MRI images. *Pattern Recogn. Lett.* 129 181–189. 10.1016/j.patrec.2019.11.019

[B38] Song J. Zheng J. Li P. Lu X. Zhu G. Shen P. (2021). An effective multimodal image fusion method using MRI and PET for Alzheimer’s disease diagnosis. *Front. Digit. Health* 3:637386. 10.3389/fdgth.2021.637386 34713109 PMC8521941

[B39] Song Q. Kasabov N. (2002). *ECM — a novel on-line, evolving clustering method and its applications.*

[B40] Tsuneki M. (2022). Deep learning models in medical image analysis. *J. Oral Biosci.* 64 312–320. 10.1016/j.job.2022.03.003 35306172

[B41] Turkson R. E. Qu H. Mawuli C. B. Eghan M. J. (2021). Classification of Alzheimer’s disease using deep convolutional spiking neural network. *Neural Process. Lett.* 53 2649–2663. 10.1007/s11063-021-10514-w

[B42] Wang G. Li W. Ourselin S. Vercauteren T. (2019). “Automatic brain tumor segmentation using convolutional neural networks with test-time augmentation,” in *Brainlesion: Glioma, multiple sclerosis, stroke and traumatic brain injuries*, eds Crimi A. (Cham: Springer International Publishing), 61–72. 10.1007/978-3-030-11726-9_6

[B43] Xiaoxue L. Xiaofan Z. Xin Y. Dan L. He W. Bowen Z. (2023). Review of medical data analysis based on spiking neural networks. *Proc. Comp. Sci.* 221 1527–1538. 10.1016/j.procs.2023.08.138

[B44] Yan W. Qu G. Hu W. Abrol A. Cai B. Qiao C. (2022). Deep learning in neuroimaging: Promises and challenges. *IEEE Signal Process. Magazine* 39 87–98. 10.1109/MSP.2021.3128348

[B45] Yang R. Yu Y. (2021). Artificial convolutional neural network in object detection and semantic segmentation for medical imaging analysis. *Front. Oncol.* 11:638182. 10.3389/fonc.2021.638182 33768000 PMC7986719

[B46] Zhu Z. Lu S. Wang S.-H. Gorriz J. M. Zhang Y.-D. (2022). DSNN: A DenseNet-based SNN for explainable brain disease classification. *Front. Syst. Neurosci.* 16:838822. 10.3389/fnsys.2022.838822 35720439 PMC9204288

