# Supplementary material for: Review of deep learning models with Spiking Neural Networks for modeling and analysis of multimodal neuroimaging data
Source: Front Neurosci. 2025 Nov 14;19:1623497. doi: 10.3389/fnins.2025.1623497 (PMC12660199; doi:10.3389/fnins.2025.1623497)
Supplement: Supplementary file 1 [file Data_Sheet_1.docx]

Supplementary Material

# Supplementary Data - Literature Review Methodology details

Preferred Reporting Items for Systematic Reviews and Meta-Analyses (PRISMA) guidelines are used for conducting this review (Moher *et al.*, 2009). The primary purpose of conducting this literature review is to analyze the trends and landscape of current advancements in deep learning models with SNN for multimodal neuroimaging data analysis. The analysis was performed using different questions, a few of which are mentioned below: -

1. What deep learning models are used?
2. What type of medical imaging modalities are mainly in use nowadays?
3. What methodology is used to extract features from neuroimaging data?
4. The advancement in SNN with integration with neuroimaging and its capabilities?

Multiple databases are used to extract articles for this study, including PubMed, IEEE Xplore, ScienceDirect, Scopus, and Nature. A few of the papers were added from other internet sources. The main keywords used are “Neuroimaging,” “Spiking Neural Networks,” and “Deep Learning.” A detailed list of all keywords used is provided in Table 1.1. Keyword searches generated numerous results, but many were heterogeneous and irrelevant; therefore, for refinement, “AND” and “OR” operators are used with double quotation marks for the keywords for better relevance. Ten years of studies are used to retrieve relevant data (ranging from 2015 to 2025) to ensure the inclusion of comprehensive coverage of knowledge and up-to-date information, as fewer relevant studies have been found in recent years. Furthermore, three additional articles were identified and included through a manual search.

| **Keywords** | **Search Terms** |
| --- | --- |
| Neuroimaging | MRI, magnetic resonance imaging, human neuroimaging, multimodal data, multimodal brain MRI, brain MRI |
| Spiking Neural Network | Spiking Neurons, SNN, Spiking Neural Network |
| Deep Learning | Deep Learning, AI, supervised learning, unsupervised learning, artificial intelligence |

Table 1.1: Keywords used in the literature review search using Boolean operators

Article selection followed PRISMA guidelines, with inclusion and exclusion determined through a three-phase process. In the identification section, all duplications are removed. During the screening phase, the title and abstract are used to identify the irrelevant articles for exclusion and inclusion. Afterward, the full text is used to extract the most relevant studies in this review. For clarity, below are the criteria to meet the inclusion of the study:

1. The article must be written in English.
2. The article must be accessible to authors/researchers.
3. The article must be peer-reviewed and published in a journal, conference, or press.
4. The article must be a research article or scientific study (not a review or commentary)
5. The article must use human data.
6. The article must be based on SNN and Neuroimaging.
